# Pathogenic *Escherichia coli* Strains Recovered from Selected Aquatic Resources in the Eastern Cape, South Africa, and Its Significance to Public Health

**DOI:** 10.3390/ijerph15071506

**Published:** 2018-07-17

**Authors:** Kingsley Ehi Ebomah, Martins Ajibade Adefisoye, Anthony Ifeanyi Okoh

**Affiliations:** 1 SAMRC Microbial Water Quality Monitoring Centre, University of Fort Hare, Alice 5700, South Africa; MAdefisoye@ufh.ac.za (M.A.A.); AOkoh@ufh.ac.za (A.I.O.); 2 Applied and Environmental Microbiology Research Group (AEMREG), Department of Biochemistry and Microbiology, University of Fort Hare, Alice 5700, South Africa

**Keywords:** *E. coli*, surface water, antibiotic-resistance gene, MARI, MARP, multidrug resistance

## Abstract

The prevalence of pathogenic microorganisms, as well as the proliferation of antimicrobial resistance, pose a significant threat to public health. However, the magnitude of the impact of aquatic environs concerning the advent and propagation of resistance genes remains vague. *Escherichia coli* (*E. coli*) are widespread and encompass a variety of strains, ranging from non-pathogenic to highly pathogenic. This study reports on the incidence and antibiotic susceptibility profiles of *E. coli* isolates recovered from the Nahoon beach and its canal waters in South Africa. A total of 73 out of 107 (68.2%) Polymerase chain reaction confirmed *E. coli* isolates were found to be affirmative for at least one virulence factor. These comprised of enteropathogenic *E. coli* 11 (10.3%), enteroinvasive *E. coli* 14 (13.1%), and neonatal meningitis *E. coli* 48 (44.9%). The phenotypic antibiogram profiles of the confirmed isolates revealed that all 73 (100%) were resistant to ampicillin, whereas 67 (91.8%) of the pathotypes were resistant to amikacin, gentamicin, and ceftazidime. About 61 (83.6%) and 51 (69.9%) were resistant to tetracycline and ciprofloxacin, respectively, and about 21.9% (16) demonstrated multiple instances of antibiotic resistance, with 100% exhibiting resistance to eight antibiotics. The conclusion from our findings is that the Nahoon beach and its canal waters are reservoirs of potentially virulent and antibiotic-resistant *E. coli* strains, which thus constitute a potent public health risk.

## 1. Introduction

Water has a function in numerous metabolic activities and is hence an essential ingredient for hydration which sustains health and sanitation, while also having industrial and agricultural applications. Thus, poor water quality has a demoralizing impact on public health, and polluted water sources can lead to waterborne disease outbreaks [1]. According to Gorde and Jadhav [2], the human population is most likely to suffer from waterborne diseases due to the use of contaminated or polluted water.

Irrespective of enormous developments in therapeutic treatment options as well as wastewater treatment facilities, waterborne infections still pose a major threat to public health worldwide [3]. These infections, caused by contaminations of surface water bodies by pathogenic microorganisms transmitted via contact with polluted water, are responsible for the illness of millions of people each year, while also causing numerous deaths [4]. The majority of these infections occur in developing nations which, in comparison with developed nations, often have less than desirable levels of sanitation, socioeconomic conditions, and public health awareness [5].

As beaches are typical spots for human recreation, they can gain a lot of patronage from both domestic and international tourists. Such recreational centers fortify development and prove to be a significant economic contribution to tourism in coastal areas [6]. Unfortunately, many beaches have been subjected to high levels of contamination in recent years [7], which is why this phenomenon has become a matter of urgency [8]. This study thus outlines the importance of maintaining a clean environment in the coastal areas and reports the discovery of pathogenic strains of bacteria exhibiting multidrug resistance.

Fecal contamination of water bodies presents severe public health issues in many countries [9] and owes the source of its threat to microbial pathogens. These are often shed by diseased humans and animals, and may be conveyed via the sewer system and agricultural run-offs [10]. In a study conducted by Okoh et al. [11], it was found that the release of ineffectively-treated effluents were the major source of enteric pathogens in aquatic environs. Due to the low monitoring of health risk that could be associated with beach water, literature has shown that potential risks may be associated with nonhuman fecal contamination [12]. *E. coli* is one of the bacteria used as an indicator organism for the monitoring of water bodies, and different strains of these bacteria are pathogenic. The pathogenicity of a specific *E. coli* pathotype is primarily determined by explicit virulent influences [13]. Globally, *E. coli* strains have been associated with human and animal diseases by means of pathogens, on the basis of their virulent elements and clinical symptoms. According to Mellata [14] and Titilawo et al. [15], *E. coli* strains can be categorized into two groups: extra-intestinal pathogenic *E. coli* (ExPEC) and intestinal pathogenic *E. coli* (InPEC). However, InPEC can also be subdivided into enteroinvasive *E. coli* (EIEC), enteroaggregative *E. coli* (EAEC), diffusely adherent *E. coli* (DAEC), enterotoxigenic *E. coli* (ETEC), enteropathogenic *E. coli* (EPEC), and enterohemorrhagic *E. coli* (EHEC). ExPEC can also be classified into neonatal meningitis *E. coli* (NMEC), uropathogenic *E. coli* (UPEC), and avian pathogenic *E. coli* (APEC) [16]. A further class known as diarrhoeagenic *E. coli* pathotypes has been proposed, such as cell-detaching *E. coli* (CDEC) although their significance remains unclear [16]. The majority of infections caused by *E. coli* are treated by using antimicrobial agents. However, the effects of some of these agents have been compromised by some types of bacteria [17]. Evidently, antimicrobial-resistant bacteria (ARB) can be released into the environment via the discarding of human and animal waste [18]. Moreover, the use of antibiotics for the treatment of infections in humans and farm animals has also been reported to cause an increase in ARB [19], and numerous antibiotics have become ineffective against their targets due to the frequent exposure of pathogens to antimicrobial agents [20,21]. The aim of this study was thus to identify and characterize the *E. coli* isolates into various pathotypes, while also determining the phenotypic resistance pattern of the confirmed isolates.

## 2. Materials and Methods

### 2.1. Description of the Study Site and Sampling Points

**Figure 1:**
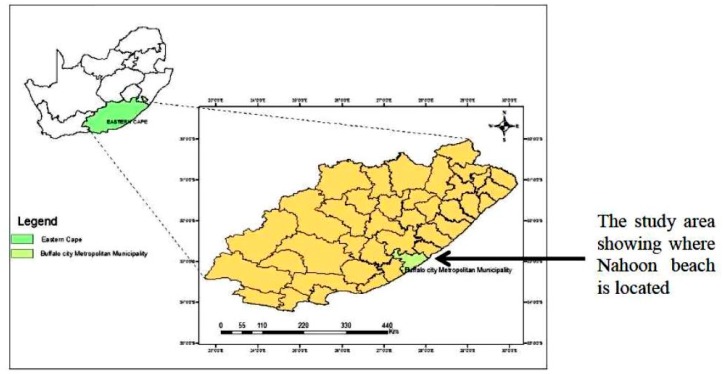
A map showing the location of the study area.

Nahoon beach and its canal are located in East London, Eastern Cape, South Africa on the coast of the Indian Ocean (geographical coordinates: 32.99° S and 27.95° E). As shown in Figure 1 below, the study area was in Buffalo City Metropolitan Municipality in the Eastern Cape, as highlighted in the map. The Nahoon canal is observed as an extension of the Nahoon River, which flows into the beach. A total of six sampling points for the beach and the canal (three points each) were mapped along the sea shore. Nahoon canal point 3 flowed into the Nahoon beach at point 1, and Nahoon canal point 2 had some domestic effluent flowing into it. There was a release of final effluent from the East Bank Reclamation Works (sited in East London, South Africa) into the Indian Ocean at Bats Cave, which is represented by sampling point 2 on the beach site in this study.

### 2.2. Sample Collection

Water samples were collected bi-weekly using Nalgene sterile bottles from 6 different sampling points along the Nahoon beach and canal for a period of twelve months, between 8:00 a.m. and 11:00 a.m. The sampling points where the water samples were collected are: canal point 1, canal point 2 (where domestic effluents flowed into canal), canal point 3 (where canal flowed into beach), beach point 1, beach point 2 (where final effluent was released into beach), and beach point 3. The samples were then transported on ice to the Applied and Environmental Microbiology Research Group (AEMREG) laboratory, University of Fort Hare, Alice within 6 h for analyses.

### 2.3. Isolation and DNA Extraction

The water samples were filtered using the membrane filtration technique, after which the filter papers were aseptically picked, placed onto *E. coli* chromogenic agar, and incubated at 37 °C for 18–24 h. After incubation, the isolates were re-streaked onto nutrient agar (NA) plates and incubated at 37 °C for 24 h. A total of 260 presumptive *E. coli* isolate colonies were picked from the NA plates, inoculated into nutrient broth, and incubated at 37 °C for 24 h. Thereafter, glycerol stock was prepared from the cultured broths, and DNA was extracted following the method of Torres et al. [22], and stored at −80 °C for further analyses.

### 2.4. Molecular Identification and Characterization of the Recovered E. coli Isolates

Molecular identification of the presumptive *E. coli* isolates targeting the *uidA* gene and the various genes of the *E. coli* pathotypes screened were determined by following the method of Titilawo et al. [15] as shown in Table 1. The PCR products were resolved in a 2% (*w*/*v*) agarose gel in 1 × TAE buffer (40 mM Tris–HCl, 20 mM Sodium Acetate, 1 mM EDTA, pH 8.5), stained with 0.5 mg mL ethidium bromide (EtBr), and visualized under the Alliance BioDoc-It System (UFH, Alice 5700, South Africa) [23,24,25]. 

### 2.5. Antimicrobial Susceptibility Pattern of the Confirmed E. coli Strains

The antimicrobial susceptibility test of the confirmed *E. coli* isolates was determined by the disc diffusion technique on Mueller Hinton agar (MHA) plates, following Clinical and Laboratory Standards Institute (CLSI) [26] guidelines. Fresh culture from the glycerol stock was streaked onto nutrient agar plates and incubated at 37 °C for 24 h. Colonies were transferred into a test tube of 5 mL of normal sterile saline, and adjusted to attain turbidity matching the 0.5 McFarland standard. The isolates were then streaked onto MHA plates, and disks infused with antimicrobial agents were dispensed onto the inoculated plates and incubated for 18 to 24 h at 37 °C. After incubation, the zones of inhibition were measured, and isolates were categorized as resistant or susceptible to the antimicrobial agents used, while those that were intermediate were considered resistant. The following eight commercial antibiotic discs: Amikacin (30 µg), ampicillin (10 µg), ceftazidime (30 µg), ciprofloxacin (5 µg), gentamicin (10 µg), norflaxacin (10 µg), tetracycline (30 µg), and trimethoprim (10 µg) were tested against the confirmed isolates.

### 2.6. Interpretation of Multiple Antibiotic-Resistance Index (MARI)

The multiple antibiotic resistance index (MAR Index) of isolates that exhibited resistance against the actions of three or more antibiotics which were tested was expressed as *x*/*y*, where *x* indicates the sum of antibiotics to which the isolate was resistant to and *y* indicates the number of antibiotics tested against the isolate. Multidrug resistance was interpreted as the display of resistance to three or more antibiotics used, whereas the MARI (multidrug antibiotic-resisted indices) of the isolates was approximated, as previously described by Krumperman [27]. The multiple antibiotic resistance index (MARI) = *w*/(*u x v*), where: *w* is the summation of antibiotics resistance scores of the isolates; *u* is the sum of antibiotics used; and *v* is the sum of isolates which resisted the antibiotics employed.

## 3. Results

### 3.1. Molecular Identification and Characterization of the Recovered E. coli Isolates

**Figure 2:**
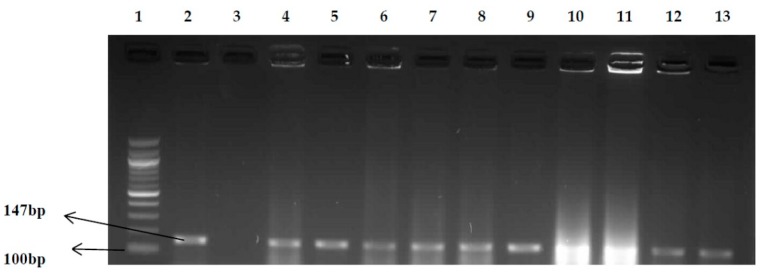
PCR products of the amplification of the *uidA* gene (*E. coli*) Lane 1: 100 bp molecular weight marker; Lane 2: positive control (*E. coli* ATCC 25922); Lane 3: negative control; Lanes 4–13: positive isolates.

A total of 260 presumptive *E. coli* isolates were obtained from the water samples following microbiological analysis. The presumptive isolates were confirmed by polymerase chain reaction techniques (PCR) targeting the *uidA* gene. Results showed that 41.2% (107/260) of the *E. coli* isolates were positive, as shown in Figure 2. The confirmed *E. coli* isolates were further characterized into different pathotypes using specific primers for each pathotype, and the result is shown in Table 2. A total number of 26 isolates belonging to the three pathotypes identified were isolated from the 3 sampling points (canal), while 47 isolates belonging to the three pathotypes identified were recovered from the 3 sampling points (beach). 

### 3.2. Antimicrobial Susceptibility Pattern of the Confirmed E. coli Pathotypes

**Figure 3:**
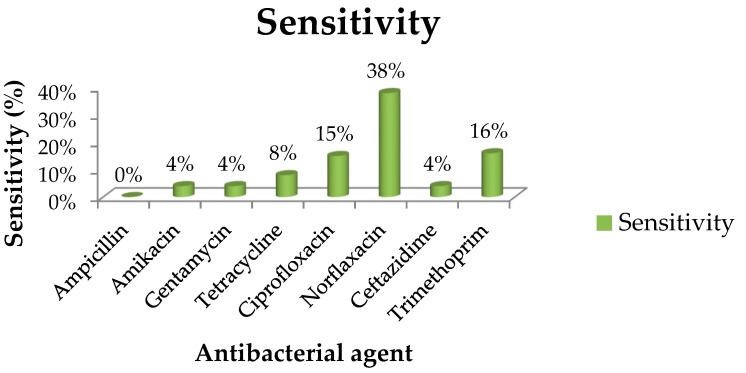
Sensitivity percentages of *E. coli* isolates to 8 antibacterial agents. The antibiotic susceptibility patterns of the isolates of the several antibiotics tested following the CLSI (Clinical and Laboratory Standards Institute) guideline [26] showed that the isolates displayed highest resistance to ampicillin (100%). The following is the order of the level of resistance exhibited against the remaining antibiotics; amikacin (96%), gentamycin (96%), ceftazidime (96%), tetracycline (92%), ciprofloxacin (85%), trimethoprim (84%), norflaxacin (62%). However, the isolates were mostly susceptible to norflaxacin.

Of the 8 test antimicrobial agents which were selected, ampicillin had the highest resistance frequency (100%). Nevertheless, amikacin and gentamycin both had quite high frequencies of 98.6% (72/73), while 70 of the strains were resistant to ceftazidime, with a frequency of 95.9% (Figure 3). About 45 strains (93% of the NMEC strains) exhibited resistance to each of ampicillin, amikacin, gentamycin, tetracycline, and ceftazidime, while 9.1% (1/11) and 91% (10/11) of the EPEC strains displayed resistance to ciprofloxacin and tetracycline respectively. For NMEC, 26 strains showed a resistance frequency of 54.2% against ciprofloxacin. Similarly, the EIEC strains demonstrated resistance ranging between 7% (1/14) and 50% (7/14) to amikacin, ampicillin, ceftazidime, ciprofloxacin, gentamycin, norfloxacin tetracycline, and trimethoprim. The results from the *E. coli* isolates which were subjected to the selected antimicrobial agents are summarized in Figure 3, which highlights all the sensitivity percentages of the isolates. 23 isolates of the various strains identified showed resistance to 8 antibiotics (19 NMEC, 3 EPEC and 1 EIEC), while 19 strains showed resistance to 7 antibiotics (5 NMEC, 4 EPEC and 10 EIEC).

### 3.3. Multiple Antibiotic-Resistance Index (MARI)

MARI of the isolates were expressed using the formula MARI = *w*/(*uxv*), as explained above. For the sampling site, the multiple antibiotic resistance index (MARI) is estimated at 0.0514.

The summation score was obtained from the total sum of MAR Index isolates from each sampling point, and the MARI value was calculated for the six sampling points.

## 4. Discussion

This study investigated the occurrence of potentially pathogenic strains of *E. coli* recovered from beach water samples. Among the total number of presumptive *E. coli* isolates screened, 73 (68%) were confirmed positive by molecular techniques, in accordance with the report of Whitman et al. [28]. The presence of these bacteria in beach water poses a high risk with regard to human contact with this water, and there are certain factors which may be responsible for the fewer number of confirmed pathogens. There is the tendency of a low survival rate due to the depth of the beach water and rapid movement of the sea waves, with a possibly high level of dilution involved [29]. Moreover, there appears to be a higher level of fecal contamination near the sea shore around the sampling points, due to the high turbidity [30,31]. In general, the presence of pathogenic *E. coli* obtained from all sampling points of this recreational facility can pose serious health risks to both tourists and bathers. A study by Tsai et al. [32] suggested that certain pedigrees of *E. coli* have adapted and become accustomed to the different aquatic milieu, and this corroborates with our results. It was observed that bacterial counts from the sampling points where wastewater was being discharged into the beach had the highest number of positive isolates during the spring season and festive period, and our findings support the report of de Carvalho and Neto [33]. Although there are many probable sources of contamination, sewage treatment plants (STP) have become a constant source of beach pollution in respect of the quality of the final effluents that are released into receiving waters [34]. 

The result from the PCR products of the 260 presumptive *E. coli* isolates screened is; 107 isolates were positive, and our result is in agreement with the report of da Costa Andrade et al. [35]. Another study by Partyka et al. [36] also identified *E. coli* from beach water, and this is also in line with our result. From the eight different *E. coli* pathotypes screened for, three groups of *E. coli* pathotypes were identified as belonging to the two categories, InPEC (EPEC and EIEC) and ExPEC (NMEC) and the frequencies of detection ranged between 10% [InPEC] and 45% [ExPEC]. The molecular identification of *E. coli* pathogens in beach water poses high risk to the people in that area who use the beach for recreational activity. This study showed that 11 (10.3%) of the 73 positive strains of *E. coli* belonged to enteropathogenic *E. coli*. A study by Byappanahalli et al. [37] has also reported the presence of EPEC strains in beach water, and this is also in line with our result. Another study by Maloo [38], carried out in India, also identified various pathotypes of *E. coli* recovered from beach water, and our report is in line with their findings. The order of the percentage of phenotypic resistance levels exhibited by the isolates against the antibiotics is as follows: ampicillin (100%), amikacin (96%), gentamycin (96%), ceftazidime (96%), tetracycline (92%), ciprofloxacin (85%), trimethoprim (84%), and norflaxacin (62%). However, the isolates were mostly susceptible to norflaxacin. A study conducted by Stoll et al. [39] in Germany and Australia revealed a high resistance rate in *E. coli* isolates recovered from surface water samples that were resistant against ampicillin and tetracycline, and our result is in accordance with their report. A high percentage of the phenotypic resistance observed in the *E. coli* isolated could either be from the origin of WWTP or agricultural waste (poultry droppings), as most of the final effluents have been discharged into water bodies [40,41].

A multiple antibiotic resistance index (MARI) was carried out in order to evaluate or assess health risks that were concomitant with the rise and spread of multidrug resistance in the environment. The MARI value of 0.2 (arbitrary) was utilized to distinguish between low and high risk to public health. In addition, a MARI value above 0.2 proposed that the pathogenic strain of bacteria originated from an environment which was highly contaminated or which had high levels of antibiotics usage [19,26]. From our study, the MARI value (0.05) obtained for the isolates was less than 0.2, signifying that the isolates originated from environments with minimal antimicrobial use. The low MARI value estimated in this study provides an opportunity for further research in this area. This could be as a result of unsuitable use of antibiotics among the populace in the study area, and any greater MARI value obtained will suggest exposure to antimicrobial pressure, which may perhaps eventually lead to an increase in multidrug resistance.

## 5. Conclusions

This research demonstrates that the aquatic environs of the Nahoon beach are potential reservoirs of pathogenic *E. coli* strains that may probably combine a high level of antimicrobial resistance. This is an indication of the pressure mount by antimicrobial usage and poses a serious public health risk to humans upon exposure, consequently, presenting a public health hazard to the people around where the beach is located.

## Figures and Tables

**Table 1:** Primer sequences of target genes and their respective amplicon sizes and PCR (polymerase chain reaction techniques) cycling conditions.

Target Strain	Target Gene	Primer Sequence (5′→3′)	Amplicon Size (bp)	PCR Cycling Condition
***E. coli***	*uidA*	F: AAA ACG GCA AGA AAA AGC AG	147	Initial denaturation of 5 min at 94 °C followed by 35 cycles, denaturation at 95 °C for 30 s, annealing at 58 °C for 1 min, extension at 72 °C for 1 min and final extension at 72 °C for 8 min
		R: ACG CGT GGT TAA CAG TCT TGC G		
**EPEC**	*eae*	F: TCA ATG CAG TTC CGT TAT CAG TT	482	Initial denaturation of 15 min at 95 °C followed by 35 cycles, denaturation at 94 °C for 45 s, annealing at 55 °C for 45 s, extension at 68 °C for 2 min and final extension at 72 °C for 5 min
		R: GTA AAG TCC GTT ACC CCA ACC TG		
		R: GGA ATC AGA CGC AGA CTG GTA GT		
**ETEC**	*lt*	F: GGC GAC AGA TTA TAC CGT GC	450	Initial denaturation of 2 min at 94 °C followed by 35 cycles, denaturation at 94 °C for 1 min, annealing at 55 °C for 1 min, extension at 72 °C for 1 min and final extension at 72 °C for 5 min
		R: CGG TCT CTA TAT TCC CTG TT		
**EAEC**	*eagg*	F: AGA CTC TGG CGA AAG ACT GTA TC	194	Initial denaturation of 15 min at 95 °C followed by 35 cycles, denaturation at 94 °C for 45 s, annealing at 55 °C for 45 s, extension at 68 °C for 2 min and final extension at 72 °C for 5 min
		R: ATG GCT GTC TGT AAT AGA TGA GAA C		
**EIEC**	*ipa*H	F: CTC GGC ACG TTT TAA TAG TCT GG	933	Initial denaturation of 2 min at 94 °C followed by 35 cycles, denaturation at 94 °C for 1 min, annealing at 55 °C for 1 min, extension at 72 °C for 1 min and final extension at 72 °C for 5 min
		R: GTG GAG AGC TGA AGT TTC TCT GC		
**DAEC**	*daa*E	F: GAA CGT TGG TTA ATG TGG GGT AA	542	Initial denaturation of 2 min at 94 °C followed by 40 cycles, denaturation at 92 °C for 30 s, annealing at 59 °C for 30 s, extension at 72 °C for 30 s and final extension at 72 °C for 5 min
		R: TAT TCA CCG GTC GGT TAT CAG T		
**EHEC**	*stx*1	F: CAG TTA ATG TGG TGG CGA AGG	384	Initial denaturation of 15 min at 95 °C followed by 35 cycles, denaturation at 94 °C for 45 s, annealing at 55 °C for 45 s, extension at 68 °C for 2 min and final extension at 72 °C for 5 min
		R: CAC CAG ACA ATG TAA CCG CTG		
**NMEC**	*ibe*A	F: TGG AAC CCC GCT CGT AAT ATA C	342	Initial denaturation of 2 min at 94 °C followed by 30 cycles, denaturation at 94 °C for 1 min, annealing at 55 °C for 1 min, extension at 72 °C for 1 min and final extension at 72 °C for 5 min
		R: CTG CCT GTT CAA GCA TTG CA		
**UPEC**	*pap*C	F: GAC GGC TGT ACT GCA GGG TGT GGC G	328	Initial denaturation of 2 min at 94 °C followed by 30 cycles, denaturation at 94 °C for 1 min, annealing at 55 °C for 1 min, extension at 72 °C for 1 min and final extension at 72 °C for 5 min
		R: ATA TCC TTT CTG CAG GGA TGC AAT A		

Source: Titilawo et al. [15].

**Table 2:** Results of *E. coli* pathotypes.

No. of Isolates Screened	Pathotype/Target Gene	No. of Positive Isolates (%)
107	EPEC/*eae*	11 (10.3%)
107	ETEC/*lt*	0
107	EAEC/*eagg*	0
107	EIEC/*ipaH*	14 (13.1%)
107	DAEC/*daaE*	0
107	EHEC/*stx1*	0
107	NMEC/*ibeA*	48 (44.9%)
107	UPEC/*papC*	0
